# The Effect of *In Vitro* Oxidative Stress on the Female Rabbit Bladder Contractile Response and Antioxidant Levels

**DOI:** 10.1155/2013/639685

**Published:** 2013-05-30

**Authors:** Lisa Malone, Catherine Schuler, Robert E. Leggett, Robert M. Levin

**Affiliations:** Stratton VA Medical Center, Albany College of Pharmacy and Health Sciences, 115 Holland Avenue, Albany, NY 12208, USA

## Abstract

*Introduction*. There are several bladder dysfunctions that are associated with oxidative stress to the urinary bladder. Two experimental models are known to cause this type of bladder damage. The first is direct oxidative damage caused by hydrogen peroxide (H_2_O_2_). The second is oxidative damage caused by ischemia followed by reperfusion (I/R). The specific aim of this study is to directly compare these two models of oxidative stress. *Methods*. Six adult female NZW rabbits were divided into two groups of three rabbits each. Eight full thickness strips from three rabbit bladders were taken for *in vitro* ischemia/reperfusion physiological analysis, while eight strips from three rabbit bladders were taken for *in vitro* H_2_O_2_ physiological analysis. All tissue was analyzed for total antioxidant activity (AA) and malondialdehyde (MDA) levels. In addition, samples of the water baths were also analyzed for AA. *Results*. *In vitro* I/R reduced the response to field stimulation (FS) to a significantly greater extent than the inhibition of the response to carbachol. *In vitro* H_2_O_2_ decreased all responses to approximately the same degree. Total AA levels at higher concentrations of H_2_O_2_ for all bath fluids were significantly higher than controls. MDA levels were significantly elevated in both models of oxidative stress.

## 1. Introduction

There are several lower urinary tract dysfunctions (LUTD) that are more prevalent in women than men including interstitial cystitis, recurrent urinary tract infections, and incontinence [1, 2]. The bladder is composed of a thick smooth muscle wall. The inner most tissue layer of the bladder wall, the mucosa (urothelium), is intimately associated with lower urinary tract function [3]. The mucosa is the first line of defense against bladder infections and the penetration of urine solutes into the bladder tissue. The glycosaminoglycan coating of the mucosal surface presents a nonadherent surface to most strains of bacteria and an impermeable barrier to urinary solutes [4–7]. We believe that incontinence, recurrent urinary tract infections, and interstitial cystitis are related directly to estrogen levels. Low estrogen can induce a significant decrease in blood flow to the bladder especially in the mucosa resulting in free radical generation (oxidative stress) and a breakdown in mucosal integrity [8–10]. The etiology of these dysfunctions involves an increase in urothelial permeability and the movement of urinary solutes such as ions and other caustic substances from the urine into the mucosa, submucosa, and muscle. This results in both the increased permeability and free radical damage to cellular and subcellular membranes including the mitochondria, sarcoplasmic reticulum, and synaptic membranes [11, 12].

Beneath the urothelium and sometimes protruding through the basal laminae is a rich network of capillaries, the suburothelial capillary plexus [13]. Our current studies, and those of others, show that urothelial function is severely compromised by both ischemia and hypoxia [3, 10, 14]. Furthermore, *in vitro* and *in vivo* rabbit models have demonstrated that bladder distention in the presence of mild hypoxia and ischemia causes increased mucosal permeability [15] and the generation of reactive oxygen species of free radicals (ROS) and reactive nitrogen species of free radicals (RNS) [16]. These factors together suggest that mucosal permeability is related to relative changes in oxygen tension and reduced blood flow. There is a significant reduction in local blood flow to the mucosa in patients with interstitial cystitis (IC) when compared with non-IC patients especially during bladder distention [17, 18]. Clinically, the symptoms of pain and urgency of patients with IC are induced by distention and relieved by voiding of the bladder. We believe this is because ischemia and hypoxia of the bladder can affect sensory nerve membranes which lead to pain activation. These data are consistent with the hypothesis that bladder distention induces relative mucosal ischemia which increases mucosal permeability and the generation of free radicals [8].

As mentioned above, alterations in estrogen levels can significantly affect bladder blood flow to the lower urinary tract as it does to the vagina and uterus. Estrogen has been shown to increase blood flow to the bladder and urethra, while low estrogen reduces blood flow below normal inducing ischemia and hypoxia [9, 10, 14]. Studies have demonstrated that ovariectomy in rabbits results in a significant decrease in blood flow to the bladder detrusor smooth muscle and mucosa. This caused increased mucosal permeability, increased free radical generation, decreased bladder and urethral contraction, and decreased bladder function [8, 19]. However, estrogen administration to ovariectomized rabbits resulted in the relief of mucosal and detrusor hypoxia, mucosal hyperplasia, and restoration of the mucosal permeability barrier as well as reduced oxidative stress [9, 10, 14]. 

We utilize two *in vitro* models of oxidative damage. In the first model of ischemia/reperfusion (I/R), isolated strips of bladder are subjected to ischemic conditions by exposure to media equilibrated with nitrogen instead of oxygen and without glucose for a period of time. The strips are then allowed to recover in the presence of oxygen and glucose to simulate reperfusion [20]. The second model involves the direct exposure of isolated bladder strips to hydrogen peroxide (H_2_O_2_). This would occur physiologically if the activity of superoxide dismutase (SOD) increased above the ability of catalase to neutralize the product of SOD activity, which is H_2_O_2_ [21].

Our specific aim of the current study is to directly compare the effects of exposure to H_2_O_2_ to that of I/R.

## 2. Materials and Methods

All methods were approved by the Institutional Animal Care and Use Committee of the Stratton VA Medical Center, Albany, NY, USA.

### 2.1. Animal Methods

Six adult female New Zealand white rabbits (approximately 3.5 kg each) were divided into two groups of three rabbits each. Each rabbit was anesthetized with 1–3% isoflurane. The bladder was rapidly removed and weighed and the rabbit euthanized with 2 mL fatal plus euthanasia fluid IV. Five strips of the bladder body and three strips of the base urethra from each bladder were taken for physiological testing. The balance of the bladder and urethra was separated by blunt dissection and frozen under liquid nitrogen and stored at −80°C for biochemical analyses.

### 2.2. Physiological Analysis

Full thickness, longitudinal strips of the bladder body, and base-urethra were mounted in individual 15 mL baths containing oxygenated Tyrode's solution at 37°C. Contractility studies on strips were performed as follows (*in vitro* I/R experiment). Each strip was set at 2 g passive tension and stimulated with field stimulation (FS: 2 Hz, 8 Hz, 32 Hz, 1 ms duration for 20 seconds with 3 minutes in between stimulations). Carbachol (20 *μ*M) was then individually administered to each strip for a 3-minute exposure followed by three washes 5 minutes apart with fresh buffer. Field stimulation was used to mimic neurotransmitter stimulation of muscle contraction through neurohumoral receptors. Carbachol is a muscarinic agonist stimulating the receptor directly without the participation of neurotransmission [8]. After control stimulations, the bath was then changed to Tyrode's solution without glucose and equilibrated with 95% nitrogen and 5% carbon dioxide for 1 hour with stimulation at 32 Hz every 5 minutes. The buffer was then changed back to normal, oxygenated Tyrode's with glucose, and the strips were allowed to recover for two hours. The strips were then stimulated as originally described and then cut, weighed, and frozen in liquid nitrogen for biochemical analyses. A sample of physiological buffer (1 mL) was taken after each stimulation and before and after the ischemic period.

Contractility studies on strips from the remaining three rabbit bladders were performed as follows (*in vitro* H_2_O_2_ experiment). The tissues were stimulated by field stimulation (FS: 2 Hz, 8 Hz, 32 V, 1 ms duration for 20 seconds with 3 minutes in between stimulations) and carbachol (20 *μ*M) individually administered to each strip for a 3-minute exposure. After carbachol, the strips were washed two times at 10-minute intervals with fresh, oxygenated Tyrode's solution. After the second wash, 0.1% H_2_O_2_ was added for a ten-minute incubation, and the tissues were stimulated again by FS and carbachol. This process was repeated for 0.2%, 0.4%, and 0.8% H_2_O_2_. A sample of physiological buffer (1 mL) was taken after each carbachol stimulation and frozen under liquid nitrogen for further biochemical analyses.

### 2.3. Biochemical Analyses

#### 2.3.1. CUPRAC Assay

Total antioxidant levels of the tissue, experimental strips, and physiological buffer samples were quantified by the cupric ion reducing antioxidant capacity (CUPRAC) assay [22, 23].

#### 2.3.2. Malondialdehyde Assay

Malondialdehyde levels of the tissue and experimental strips were quantified using a TBA-based assay [24, 25].

### 2.4. Statistical Analyses

One-way analysis of variance was used to determine if significant differences were present among the groups, and the Tukey test was used to compare individual groups. A  *P* < 0.05 was required for statistical significance.

## 3. Results

**Figure 1:**
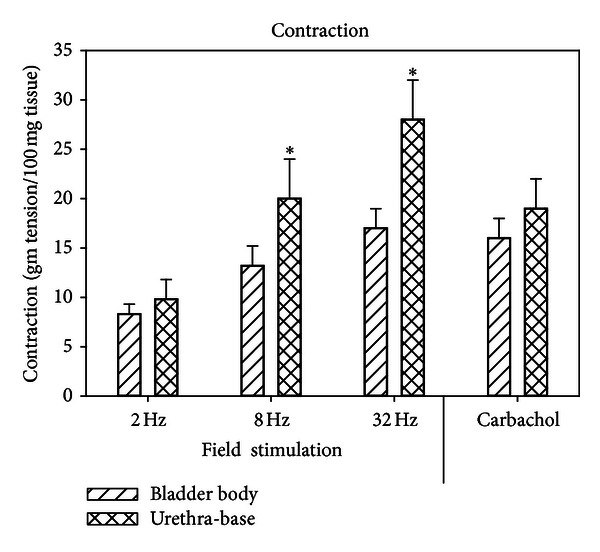
Contractile responses to field stimulation and carbachol for the bladder body and base-urethral strips. Each bar is the mean ± SEM of 6 individual rabbits. * = significantly different from bladder body, *P* < 0.05.

**Figure 2:**
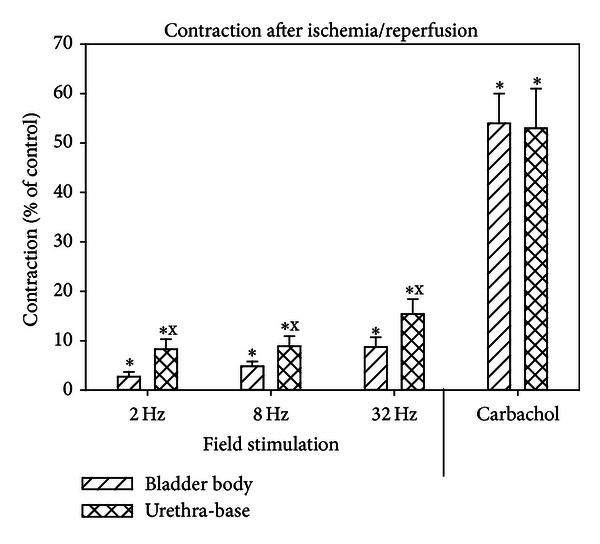
Effect of ischemia/reperfusion on the contractile responses to field stimulation and carbachol for the bladder body and base-urethral strips. Each bar is the mean ± SEM of 3 individual rabbits. * = significantly different from control; x = significantly different from bladder body, *P* < 0.05.

The contractile responses of the bladder body and base-urethra tissue are presented in Figure 1. The responses of the base-urethral strips to 8 and 32 Hz were significantly higher than the responses of the bladder body strips for each stimulation.  Figure 2 shows the contractile responses after recovery from ischemia/reperfusion (I/R) as the percent of the control response (before I/R). Whereas the recovery of the strips to field stimulation reached only 20% of the control response, the recovery of carbachol reached 60% of control. The recovery of the base-urethral strips was significantly higher to field stimulation than were the bladder body strips.

**Figure 3:**
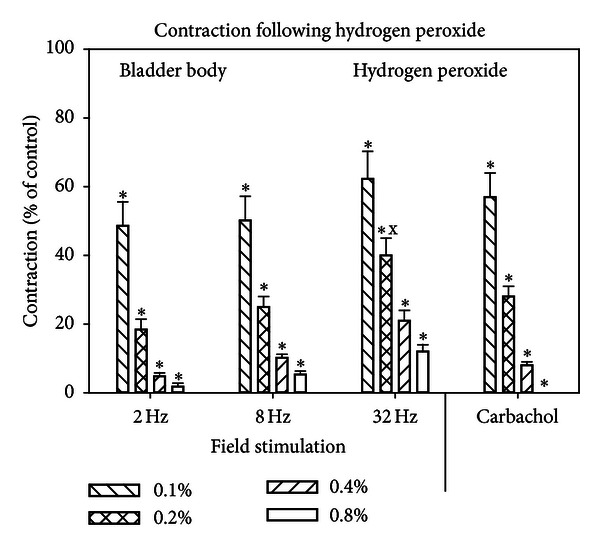
Effect of hydrogen peroxide on the contractile responses to field stimulation and carbachol for the bladder body. Each bar is the mean ± SEM of 3 individual rabbits. * = significantly different from control; x = significantly different from the effects of 0.2% hydrogen peroxide on other forms of stimulation, *P* < 0.05.

**Figure 4:**
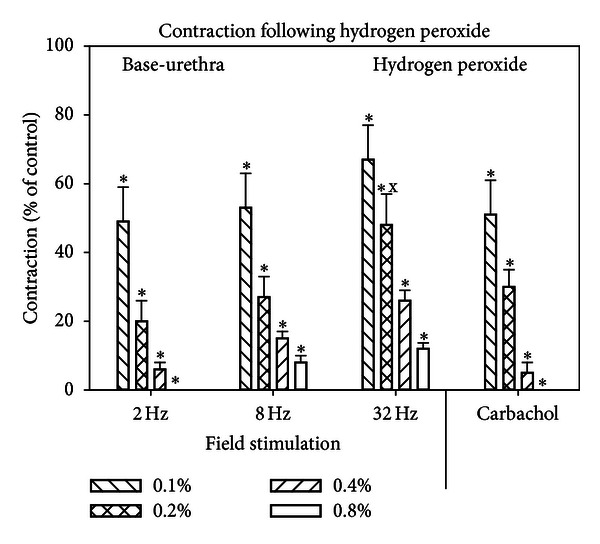
Effect of hydrogen peroxide on the contractile responses to field stimulation and carbachol for the bladder base-urethra. Each bar is the mean ± SEM of 3 individual rabbits. * = significantly different from control; x = significantly different from the effects of 0.2% hydrogen peroxide on other forms of stimulation, *P* < 0.05.

Figures 3 and 4 show the responses of the bladder body (Figure 3) and base-urethra (Figure 4) to H_2_O_2_. H_2_O_2_ produced a progressive decrease to all forms of stimulation to approximately the same level for both tissues except for the 32 Hz stimulation which was less sensitive to H_2_O_2_ than all other forms of stimulation. Interestingly, carbachol was more sensitive to hydrogen peroxide than it was to I/R. 

**Figure 5:**
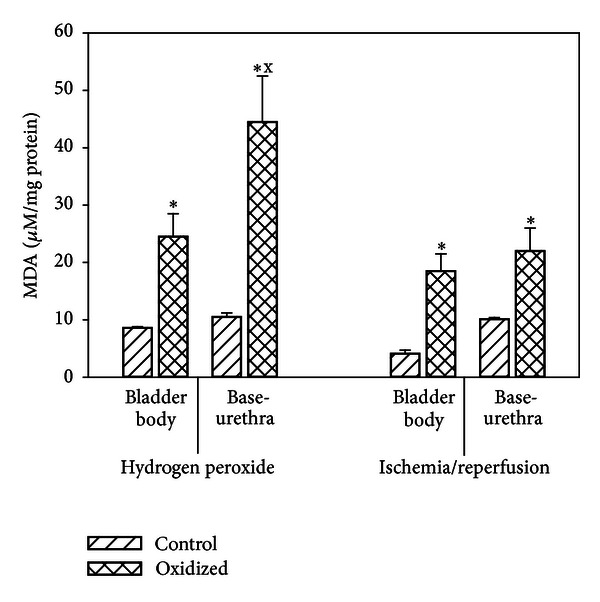
Effect of hydrogen peroxide and ischemia/reperfusion on MDA concentrations. Each bar is the mean ± SEM of 3 individual rabbits. * = significantly different from control; x = significantly different from bladder body, *P* < 0.05.

Malondialdehyde (MDA) is a biomarker for peroxidative damage to lipids [24, 26, 27]. In both *in vitro*  I/R and H_2_O_2_ experiments, MDA levels were significantly elevated in the experimental strips compared to control tissue with higher levels seen in the oxidized base-urethra compared to the bladder body. Significantly, higher levels were seen in the H_2_O_2_ experiments than in the I/R experiments (Figure 5).

**Figure 6:**
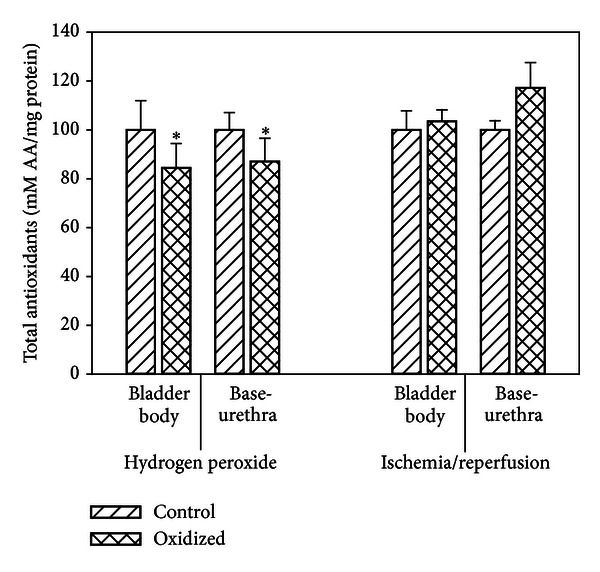
Total antioxidants of bladder body and base-urethra before and after hydrogen peroxide and ischemia/reperfusion. Each bar is the mean ± SEM of 3 individual rabbits. * = significantly different from oxidized body and base-urethra following ischemia/reperfusion, *P* < 0.05.

The total antioxidant activity levels (AA) for all tissue in both experiments did not differ significantly. The data has been normalized to control = 100 to allow for a better view of the effect of oxidation on the AA (Figure 6). It should be noted however that AA of the H_2_O_2_ oxidized tissues were significantly lower than the AA of the oxidized I/R tissues. 

**Figure 7:**
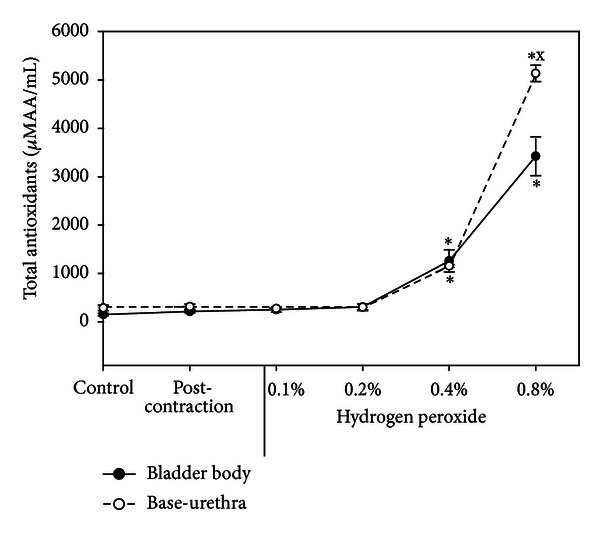
Total antioxidants in the bath water following hydrogen peroxide. Each symbol is the mean ± SEM of 3 individual rabbits. * = significantly different from control, x = significantly different from bladder body, *P* < 0.05.

**Figure 8:**
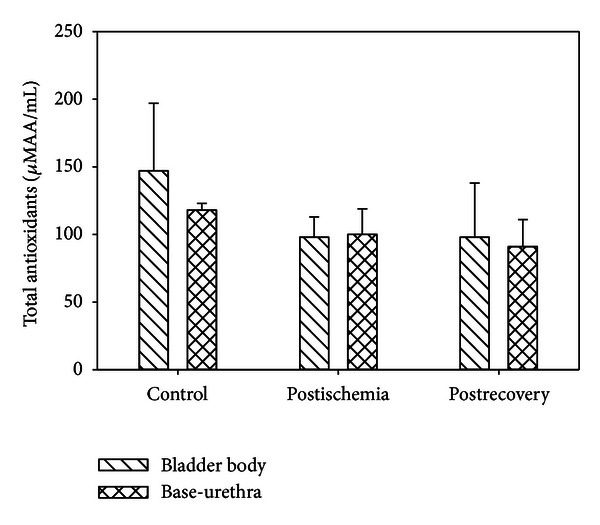
Total antioxidants in the bath water following ischemia/reperfusion. Each symbol is the mean ± SEM of 3 individual experiments.

The AA of the organ bath buffer were elevated in the *in vitro* H_2_O_2_ experiment when the concentration of H_2_O_2_ was increased above 0.2%. The AA at 0.4% and 0.8% H_2_O_2_ for both bladder body and urethra-base were significantly higher than the control levels (Figure 7). The AA of the organ bath buffers from the I/R experiment were not significantly higher than control (Figure 8).

## 4. Discussion

The specific aim of the current study was to directly compare the two models of *in vitro* oxidative damage. The data from our contractility studies revealed that the two models have very different effects on the contractile responses to FS and to carbachol. The response to FS was inhibited to a significantly greater degree than to carbachol in the I/R experiment than the H_2_O_2_ experiment. H_2_O_2_ produced a similar decrease in bladder tissue contractility response to both carbachol and field stimulation. The significance of this is that I/R targets the synapse and synaptic transmission to a greater extent than it does the muscarinic receptor or the cell membrane, whereas H_2_O_2_ targets the synapse equally to the muscarinic receptor [28].

Furthermore, our biochemical analyses revealed that MDA levels were significantly higher for both bladder body and base-urethra in the hydrogen peroxide experiment when compared to those of the I/R experiment. MDA is a biomarker for oxidative damage and a product of fatty acid lipid peroxidation induced by reactive oxygen species [24, 26, 27]. Interestingly, in the H_2_O_2_ experiment the generation of MDA in the base-urethra tissue was significantly higher than the body tissue. In the I/R experiment, the MDA response was approximately the same for the two tissues showing another difference in the responses to the two forms of oxidative stress that were utilized.

Since the H_2_O_2_ is added to the organ bath in the contractile studies, the intracellular oxidative effects cannot be directly compared to the I/R induced oxidative stress. However, H_2_O_2_ is a naturally occurring oxidative compound. Physiologically, superoxide dismutase (SOD) will metabolize superoxide to hydrogen peroxide (H_2_O_2_), which in turn will be converted to O_2_ and H_2_O by catalase. Pathological conditions can significantly alter the concentrations of SOD and catalase and thus result in the accumulation of H_2_O_2_ which is a strong oxidant capable of causing oxidative damage [21]. Both models have been utilized in experimental models of oxidative stress in the bladder [29, 30].

It should also be noted that at higher concentrations of H_2_O_2_ (above 0.2%), total antioxidant levels (AA) were elevated in the organ bath buffers, while levels in the I/R experiments remained constant. The fact that the AA of the oxidized tissues from the H_2_O_2_ experiments was significantly higher than the AA of the oxidized tissue from the I/R experiment is consistent with the leakage of the AA from the tissue to the bath in the H_2_O_2_ experiments. Consistent with this is the demonstration that in the H_2_O_2_ experiments, the AA concentrations of the oxidized tissues were significantly lower than the oxidized tissues of the I/R experiment.

## 5. Conclusions

Both forms of *in vitro* oxidative stress resulted in significant decreases in contractility and increased lipid peroxidation. However, the results clearly demonstrate that I/R and H_2_O_2_ act significantly differently on the bladder and thus should not be used interchangeably. In general, since *in vivo* models of bladder dysfunction such as partial outlet obstruction inhibit field-stimulated contraction to a significantly greater degree than carbachol and KCl inhibit, it shows that *in vitro * ischemia/reperfusion would be closer to obstructive bladder dysfunction than H_2_O_2_.
